# Self-organization of mesoscopic silver wires by electrochemical deposition

**DOI:** 10.3762/bjnano.5.142

**Published:** 2014-08-15

**Authors:** Sheng Zhong, Thomas Koch, Stefan Walheim, Harald Rösner, Eberhard Nold, Aaron Kobler, Torsten Scherer, Di Wang, Christian Kübel, Mu Wang, Horst Hahn, Thomas Schimmel

**Affiliations:** 1 Institute of Applied Physics and Center for Functional Nanostructures (CFN), Karlsruhe Institute of Technology (KIT), 76128 Karlsruhe, Germany; 2 Institute of Nanotechnology (INT), Karlsruhe Institute of Technology (KIT), 76021 Karlsruhe, Germany; 3 Institute of Materials Physics, University of Muenster, 48149 Muenster, Germany; 4 Institute for Materials Research I (IMF I) Karlsruhe Institute of Technology (KIT), 76021 Karlsruhe, Germany; 5 Joint Research Laboratory Nanomaterials (KIT and TUD), Technische Universität Darmstadt (TUD), Petersenstr. 32, 64287 Darmstadt, Germany; 6 Karlsruhe Nano Micro Facility (KNMF), Karlsruhe Institute of Technology (KIT), Hermann-von-Helmholtz-Platz 1, 76344 Eggenstein-Leopoldshafen, Germany; 7 National Laboratory of Solid-State Microstructures, Nanjing University, Nanjing 21009, China; 8 Helmholtz Institute Ulm Electrochemical Energy Storage, Albert-Einstein-Allee 11, 89081 Ulm, Germany; 9 Herbert Gleiter Institute of Nanoscience, NUST, Nanjing 21009, China

**Keywords:** crystal growth, electrochemistry, electrodeposition, mesowires, nanoelectrochemistry, nanowires, self-organization, silver nanowires, silver nitrate, stability

## Abstract

Long, straight mesoscale silver wires have been fabricated from AgNO_3_ electrolyte via electrodeposition without the help of templates, additives, and surfactants. Although the wire growth speed is very fast due to growth under non-equilibrium conditions, the wire morphology is regular and uniform in diameter. Structural studies reveal that the wires are single-crystalline, with the [112] direction as the growth direction. A possible growth mechanism is suggested. Auger depth profile measurements show that the wires are stable against oxidation under ambient conditions. This unique system provides a convenient way for the study of self-organization in electrochemical environments as well as for the fabrication of highly-ordered, single-crystalline metal nanowires.

## Introduction

Nanoscale and mesoscale metal wires have attracted considerable attention due to their potential application in new electronic, sensor, and optical devices [1–10]. Furthermore, metallic nanowires and -contacts play a key role as leads and contacts for contacting molecules in the field of molecular electronics (for a collection of recent work see [11–19]). Silver wires, in particular have been the focus of research due to their excellent electric and optical properties [4–6,9–10,20]. For example, itinerant electrons in silver wires can strongly interact with incident electromagnetic waves at specific frequencies and induce a collective resonant absorption on the surface known as surface plasmon resonance [21]. Because of this feature, noble metals can serve as plasmon waveguides [22–23]. Especially, single-crystalline metallic materials are preferred in order to reduce the loss in transmitting signals. Therefore, the fabrication of microscopic building blocks, such as single-crystalline silver wires, is a crucial step towards the implementation of nanodevices and represents a significant challenge in nanoscale science. There are various existing methods to fabricate mesoscale metallic wires: Electron beam lithography is a precise and well-controlled method, yet for larger numbers of wires rather expensive and time consuming. Electrochemically oxidized anodic alumina membrane (AAM) templates are also often used to fabricate metallic nanowires [24]. Yet the AAM-mediated nanowires are often inhomogeneous in morphology, poly-crystalline in structure, and fragile in their mechanical properties. This is also true for nanowires fabricated with the tip of an atomic force microscope used as a mechano-electrochemical pen [25]. As reported recently, this technique allows to fabricate predefined metallic structures on surfaces with nanoscale resolution, which, however cannot be fabricated as freestanding wires [25–26].

Here we report a unique method to long, straight, and single-crystalline mesoscopic silver wires by electrochemical deposition in the potentiostatic mode without the need to use any templates, surfactants or additives. At the same time, our method has the advantages of high deposition rate, low reaction temperature, and low cost which are traditionally associated with electrochemical deposition techniques [27].

## Results and Discussion

**Figure 1:**
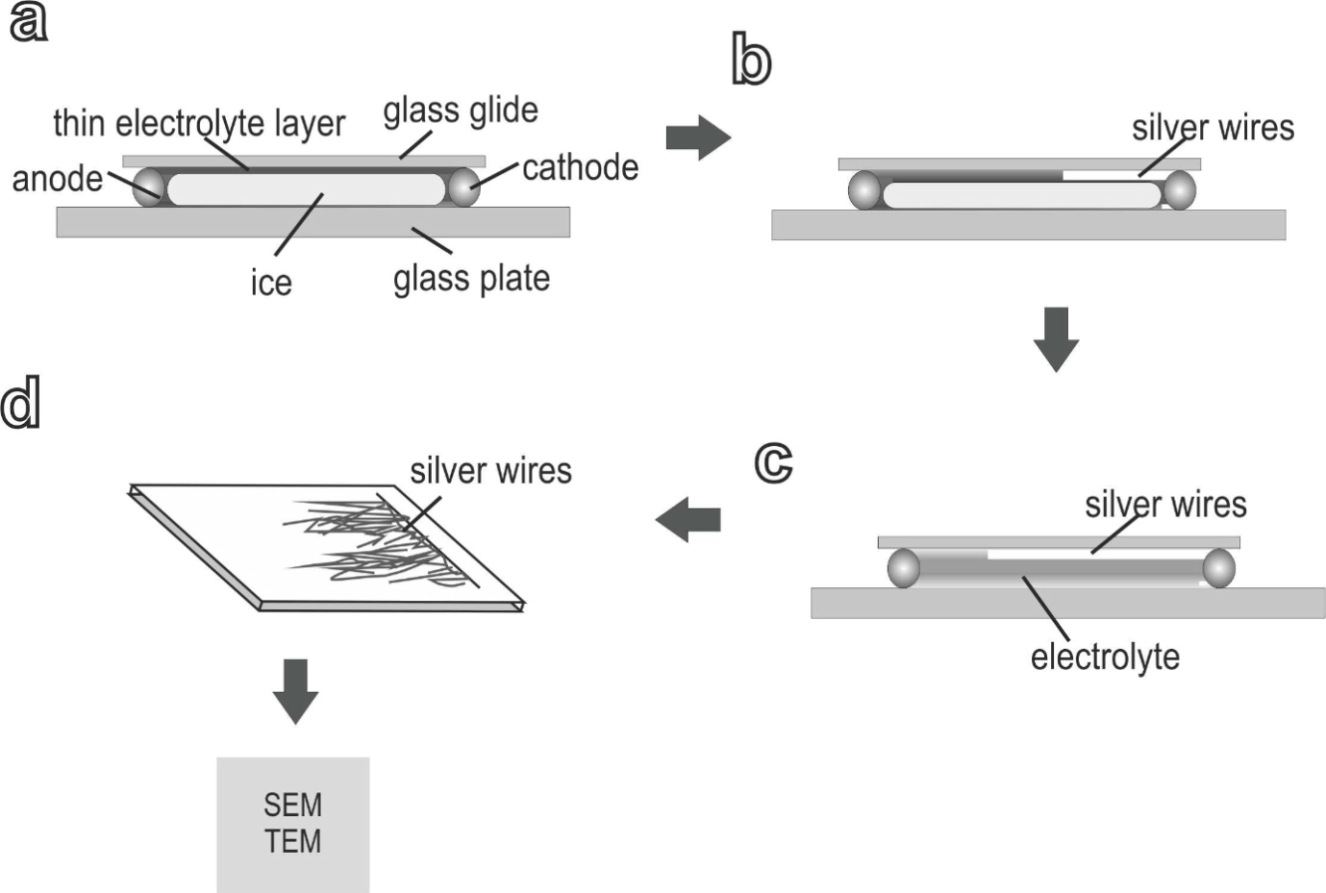
Schematic diagrams of the experimental procedure. (a) By slowly freezing the silver nitrate electrolyte a thin aqueous layer of electrolyte forms between the glass slide and the ice. The concentration of electrolyte is higher than the initial concentration due to the segregation effect. (b) Applying a constant voltage across the two electrodes let the silver grow from the cathode into the electrolyte. (c) Cooling is stopped and the temperature rises after deposition. After melting the ice the silver wires can be taken out of the deposition cell and rinsed with deionized water (d). After drying they are ready for further TEM- and SEM-analysis.

The fabrication process of the mesoscopic silver wires is summarized in Figure 1. It is similar to that described in our previous work [28–31]. Before electrodeposition, the electrolyte in the deposition cell is carefully solidified by lowering the temperature to a preset value, which is usually just below the freezing point of the electrolyte. Due to the segregation effect, AgNO_3_ is partially expelled from the ice of the electrolyte during solidification [32]. As a consequence, the concentration of aqueous AgNO_3_ electrolyte in the deposition cell increases. When equilibrium is reached, a thin layer of concentrated AgNO_3_ electrolyte is formed between frozen electrolyte and the glass plates of the deposition cell (Figure 1a).

Thereafter, a constant voltage is applied across the two electrodes, and deposits first nucleate from the cathode, grow laterally into the aqueous electrolyte, and form aligned wires which grow towards the anode (Figure 1b). It takes several minutes before the deposit occupy an area of about 0.5 cm^2^. When electrodeposition is finished, the temperature is increased to melt the ice (Figure 1c). The wire deposits stack on the glass substrate and can be easily taken out of the electrodeposition cell for further examinations (Figure 1d). The thickness of the electrolyte layer can be roughly estimated by two methods: 1. the thickness of the deposits piled on the substrate [28,31] and 2. by measuring the electric resistance across the cell [33]. Using method 2 the authors showed, that the thickness of the CuSO_4_ electrolyte layer can be tuned from 100 nm to 1.4 µm by changing the temperature from −8 °C to −1 °C [33]. In the experiments presented here the thickness of the layer was in the range of several micrometers based on the observed thickness of the deposition.

**Figure 2:**
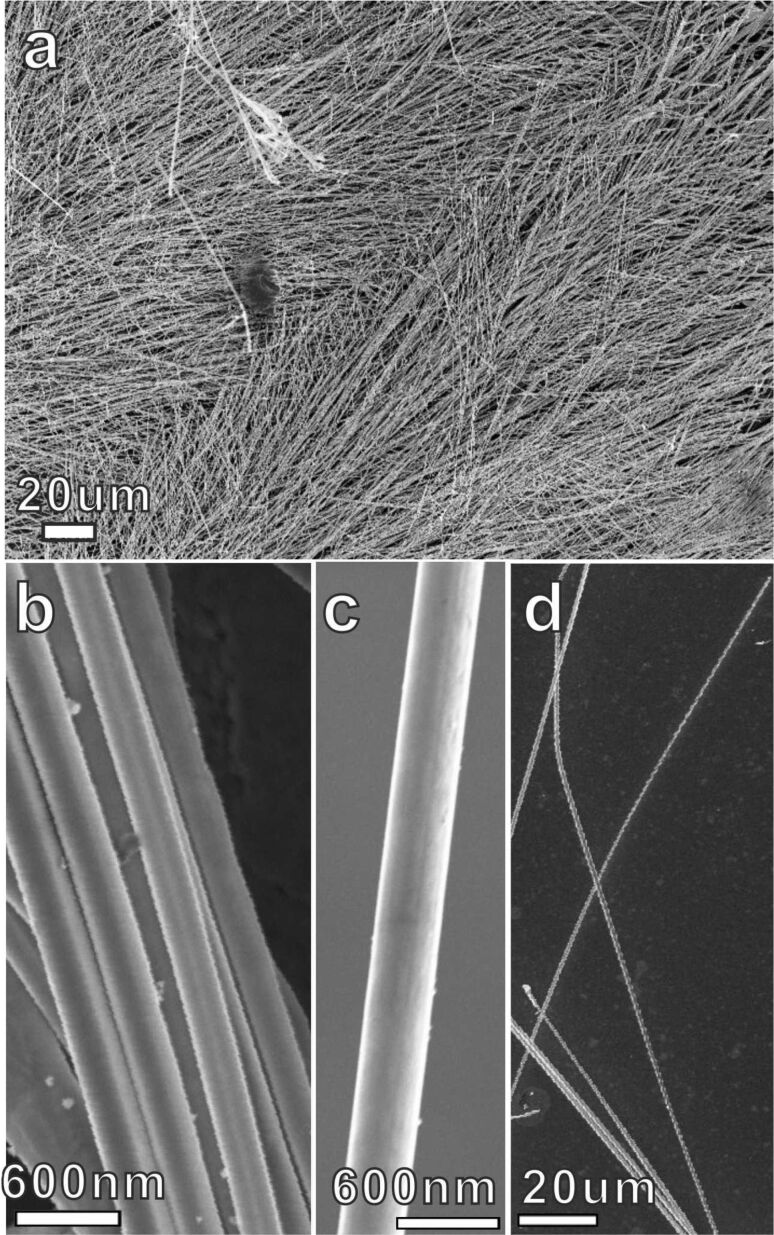
SEM images of silver wires: (a) Overview: low-magnification image. (b) Zoom-in of (a). (c) Image of one silver wire, illustrating the homogeneous thickness and smooth surface. (d) Image of several long silver wires.

Scanning electron microscopy (SEM) shows that the silver wires are growing parallel to the glass substrate (Figure 2). Bunches of silver wires initially nucleate on the cathode and propagate laterally on the glass substrate parallel to the local electric field. Eventually silver wires cover the glass substrate and pile up like log stacks. The overall morphology of the deposits exhibits bunch- and tree-like structures, as shown in Figure 2a. Detailed features of the silver wires are illustrated in Figure 2b and 2c, where the wires are straight uniform in diameter and exhibit a smooth surface. The diameter of the wires ranges from about 150 nm to 600 nm. Preliminary results show a strong influence of the applied voltage on the diameter of the wires.

Several long wires are shown in Figure 2d. Sometimes the length of the wires exceeds 150 µm. As illustrated in Figure 2, the deposits macroscopically consist of long and straight wires, occasionally with regular side branches. Microscopically, straight silver wires are often aligned and form ordered arrays. In our experiments, the silver wires are robust and can be taken out of the cell together with the substrates without being damaged. They can be removed from the substrate and subsequently rearranged on other substrates.

The microstructure and chemical composition of the silver wires were analyzed by transmission electron microscopy (TEM) and energy dispersive X-ray (EDX) spectroscopy.

**Figure 3:**
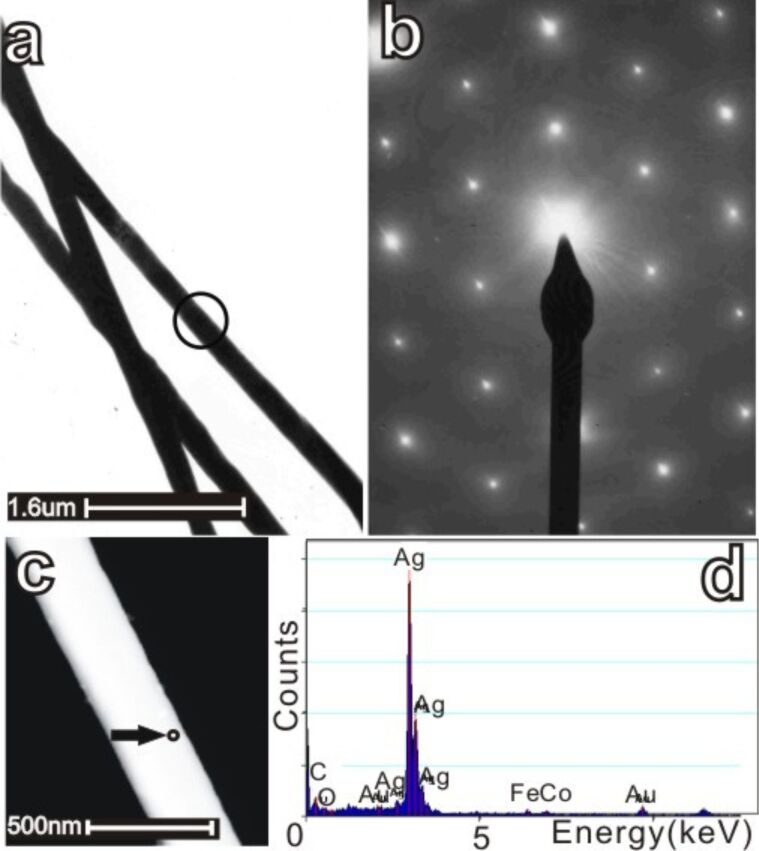
TEM analysis of thin silver wires and corresponding EDX information. (a) Bright-field image of typical silver wires with the axes which should be oriented in [112] direction and (b) the corresponding SAED patterns recorded from the marked region, the growth direction is mostly like [112]. The zone axis is <111>. (c) HAADF-STEM image of a silver wire and (d) the corresponding EDX spectrum taken at the labelled position on the wire of Figure 3c. Strong silver element peaks can be identified. Very weak gold, iron, carbon, and cobalt peaks were also found. Gold and carbon come from the substrate. The cobalt and iron signal comes from the pole pieces of the objective lens; no peak can be attributed to sulfur, nitrogen or oxygen, the latter demonstrating that the silver wires are not oxidized at ambient conditions in air.

Figure 3a shows a typical bright-field TEM image of silver wires and the corresponding selected area electron diffraction (SAED) pattern (Figure 3b). The SAED patterns demonstrate a distinct single-crystalline feature. The data show that the growth direction of the wire is perpendicular to the [111] direction, along the [112] direction. Figure 3c is a scanning TEM (STEM) image of a silver wire. Figure 3d shows the EDX spectrum collected from the marked region in Figure 3c. Strong silver element peaks are identified, together with very weak gold, iron, carbon, and cobalt peaks. Gold and carbon signals most likely come from the carbon-covered gold TEM grid, on which the sample has been deposited. Cobalt and iron most likely come from the pole pieces of the objective lens. Oxygen and sulfur signals are not observed. Therefore, one can conclude that the silver wires grown in the experiments presented here are chemically pure.

The application of silver wires as building blocks for microelectronics requires good chemical stability, especially the stability against oxidation under ambient conditions. For most metallic microstructures, however, stability against oxidation is a challenge. The reason is that reducing the length scale means increasing the surface/volume ratio and thus induces more instability in comparison with bulk systems. Therefore, the aging of the silver wires was investigated by scanning Auger micro spectroscopy (SAMS).

**Figure 4:**
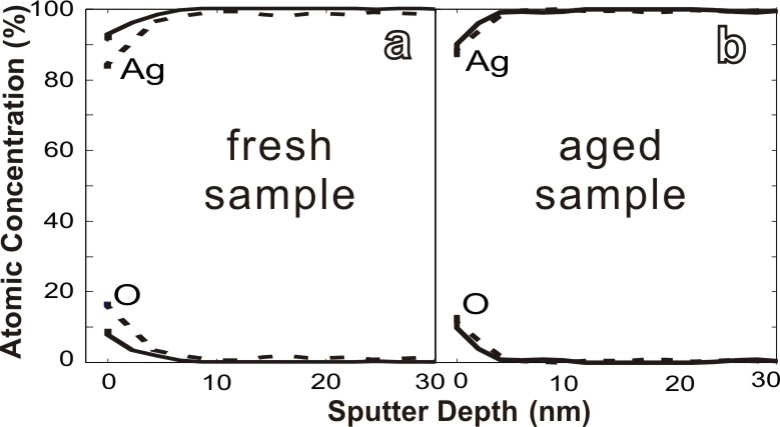
Auger depth profile curves of freshly prepared and aged silver wires. The full and dotted curves correspond to data obtained from two different wires. The relative atomic concentration is plotted versus the sputter depth; (a) freshly-prepared sample taken out of the electrodeposition cell and washed by deionized water and immediately investigated by SAMS. (b) Sample aged by exposure to ambient conditions for four months. Freshly-prepared and aged samples show similar depth profiles, indicating that the single-crystalline silver wires are stable under ambient conditions on this time scale.

Figure 4 shows the element depth profiles of silver wires, which were exposed to ambient conditions for different times. The profiles are determined by Auger measurements between several short sputtering steps. The left diagram shows data obtained from a freshly-prepared sample (exposed to ambient air for less than one hour) and the right one the data of an aged sample that had been exposed to ambient conditions for four months. Comparing the two depth profiles, no significant differences can be identified. As illustrated in Figure 4, in a depth of only 5 nm, the concentration of the oxide already virtually approaches zero. The thickness of the oxide layers did not change significantly during the aging process and the oxide layer is not more than approx. 5 nm thick. Therefore, Figure 4 indicates that the silver wires are stable against oxidation under ambient conditions. Silver typically corrodes under ambient conditions by silver sulfidation with hydrogen sulfide (H_2_S) and/or carbonyl sulfide (OCS) in the atmosphere, with this phenomenon being amplified by water and oxygen [34]. The origin of the stability of our structures is the subject of an ongoing study.

It is interesting to consider the mechanism for electrochemical self-organization of such long, smooth silver wires, bearing in mind, that the growth rate of the wires is of the order of 10 μm/s in our experiments and that the entropy of interfacial phase transition for silver is small. The faces tend to be roughened [34] and the surfaces of the wires are rounded without obvious faces. This may indicate that the surface is rough on atomic scale. It is known, that the (112) surface energy is relatively high and that the growth speed of [112] is faster than that of other facets [35]. Hence elongated silver wires are generated due to the anisotropy in growth rate. However, anisotropy in growth rate cannot guarantee for the formation of smooth silver wires with homogeneous diameter as that shown in Figure 2b,c. The point is that the side faces of the wires are rough on the atomic scale, and should possess linear growth kinetics. Thus, any small driving force should make the side faces to grow. If this would be the case, then we would observe conical silver wires instead of iso-diameter ones. We suggest that the formation of uniform silver wires in our system is due to the unique geometric restriction of the deposition cell and the screening effect in Laplacian field [36–37]. According to Chazalviel, the cation concentration behind the growing front decreases dramatically in a two-dimensional electrodeposition system [37]. Therefore, active deposition takes place only on the growth front, and growth in those parts behind the growth front is virtually stopped. In a thick electrodeposition cell, this condition cannot be realized easily because of convection and migration of the ions in the electric field. Our thin film electrodeposition system is closer to an ideal two-dimensional growth system, which helps to explain the low diameters of the silver wires.

**Figure 5:**
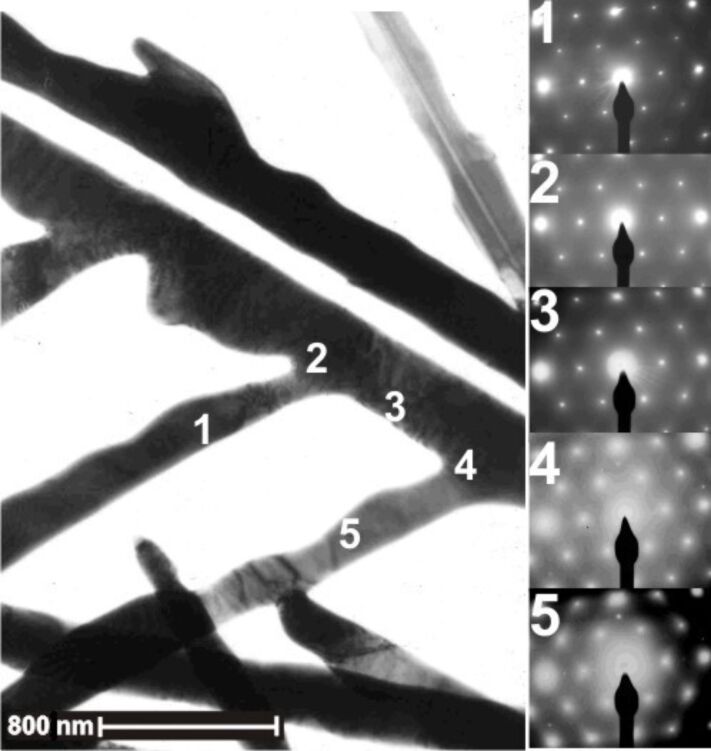
TEM analysis of thin comb-like silver dendrites (dark areas in the image left) and corresponding SAED patterns. Patterns (1)–(5) (right) were recorded at different spots marked by the corresponding number in the TEM image (left). The zone axis is <111>. This suggests that the comb structure is single-crystalline. All SAED patterns display the same hexagonal pattern. This suggests that the comb structure is single- crystalline. Each branch should grow along one of the <112> directions.

The growth mechanism of the silver wires can be explained in the following model: Initial silver wires nucleate on the cathode and grow towards the anode, presumably with [112] as the preferred growth direction. Behind the growth front the wires do not increase their diameter due to the depletion effect. These two factors allow the wires, once they are initiated along the direction of local concentration gradient, to develop into homogeneous, non-branching wires, as shown in Figure 2b,c. However, if the axis of the wire deviates from the local concentration gradient, e.g., if in the initial direction of the axis of the wire is not perpendicular to the cathode (anode), sidebranches will be triggered. Since the sides of the silver wires are rough, there is no significant energy barrier to prevent the generation of sidebranches. When this sidebranching mechanism works, the sidebranches should develop on only one side of the wires, that is, from the side facing the cation supply. Indeed such comb-like structures were observed in our experiments, too. As illustrated in Figure 5, side branching takes place only on one side of the wire and forms a 60 degree angle with respect to the main stem. Electron diffraction indicates that the side branches maintain the same crystallographic direction.

These observations confirm our understanding of the silver wire growth and support the nutrient-supply-flux induced side branching mechanism in silver wire growth. This branching mechanism is valid when the wire growth has very strong anisotropy and the side faces of the wire are rough.

## Conclusion

We have reported a novel technique for fabricating single-crystalline silver wires by electrochemical deposition, without introducing templates, additives and surfactants. The simple experimental setup and the wide range of control parameters make this approach a versatile and simple pathway to fabricate metal meso- and nanostructures. This unique system provides a convenient way to investigate fundamental processes of self-organization in electrochemical environments. Our results are pointing the way to a controlled fabrication of highly-ordered, single-crystalline metal nanowires, opening perspectives, e.g., for on-chip electrical connectors, sensors as well as photonic and plasmonic devices.

## Experimental

Electrodeposition was carried out in a cell with two parallel electrodes made of silver wires (99.99%). The electrolyte solution was prepared with AgNO_3_ (99.8%) and deionized water. The concentration of the solution was 0.05 M.

Scanning electron microscopy (SEM) was performed with a Gemini 2 system from Zeiss (LEO). The scanning Auger micro spectroscopy (SAMS) investigations were done with a PHI 680 AUGER NANOPROBE UHV system with 15 nm resolution and depth profile equipment. Transmission electron microscopy (TEM) investigations were performed with a Philips Tecnai F20 ST system operated at 200 kV. EDX analyses were carried out in STEM (scanning transmission electron microscopy) mode in order to measure nanometer-scale samples (1 nm spot size for the EDX measurements presented in this article). STEM micrographs were acquired using an HAADF (High-Angle Annular Dark-Field) detector.

## References

[1] Lieber C M (2001). Sci Am.

[2] Chen J, Wiley B J, Xia Y (2007). Langmuir.

[3] Kuchibhatla S V N T, Karakoti A S, Bera D, Seal S (2007). Prog Mater Sci.

[4] Rycenga M, Cobley C M, Zeng J, Li W, Moran C H, Zhang Q, Qin D, Xia Y (2011). Chem Rev.

[5] Hartland G V (2011). Chem Rev.

[6] Dawson K, Strutwolf J, Rodgers K P, Herzog G, Arrigan D W M, Quinn A J, O’Riordan A (2011). Anal Chem.

[7] Wild B, Cao L, Sun Y, Khanal B P, Zubarev E R, Gray S K, Scherer N F, Pelton M (2012). ACS Nano.

[8] Mann S A, Garnett E C (2013). Nano Lett.

[9] Garnett E C, Cai W, Cha J J, Mahmood F, Connor S T, Greyson Christoforo M, Cui Y, McGehee M D, Brongersma M L (2012). Nat Mater.

[10] van de Groep J, Spinelli P, Polman A (2012). Nano Lett.

[11] May F, Wegewijs M R, Hofstetter W (2011). Beilstein J Nanotechnol.

[12] Hong W, Valkenier H, Mészáros G, Manrique D Z, Mishchenko A, Putz A, García P M, Lambert C J, Hummelen J C, Wandlowski T (2011). Beilstein J Nanotechnol.

[13] Perrin M L, Martin C A, Prins F, Shaikh A J, Eelkema R, van Esch J H, van Ruitenbeek J M, van der Zant H S J, Dulić D (2011). Beilstein J Nanotechnol.

[14] Todorov T N, Dundas D, Paxton A T, Horsfield A P (2011). Beilstein J Nanotechnol.

[15] Obermair C, Kuhn H, Schimmel T (2011). Beilstein J Nanotechnol.

[16] Strange M, Thygesen K S (2011). Beilstein J Nanotechnol.

[17] Nakashima S, Takahashi Y, Kiguchi M (2011). Beilstein J Nanotechnol.

[18] Lü J-T, Gunst T, Hedegård P, Brandbyge M (2011). Beilstein J Nanotechnol.

[19] Solomon G C, Bergfield J P, Stafford C A, Ratner M A (2011). Beilstein J Nanotechnol.

[20] Gaynor W, Burkhard G F, McGehee M D, Peumans P (2011). Adv Mater.

[21] El-Sayed M A (2001). Acc Chem Res.

[22] Barnes W L, Dereux A, Ebbesen T W (2003). Nature.

[23] Wang W, Yang Q, Fan F, Xu H, Wang Z L (2011). Nano Lett.

[24] Nishizawa M, Menon V P, Martin C R (1995). Science.

[25] Obermair C, Wagner A, Schimmel T (2011). Beilstein J Nanotechnol.

[26] Obermair C, Kress M, Wagner A, Schimmel T (2012). Beilstein J Nanotechnol.

[27] Wang Z L (2000). Characterization of nanophase materials.

[28] Wang M, Zhong S, Yin X-B, Zhu J-M, Peng R-W, Wang Y, Zhang K-Q, Ming N-B (2001). Phys Rev Lett.

[29] Zhong S, Koch T, Wang M, Scherer T, Walheim S, Hahn H, Schimmel T (2009). Small.

[30] Zhong S, Wang D, Koch T, Wang M, Walheim S, Schimmel T (2010). Cryst Growth Des.

[31] Zhong S, Wang Y, Wang M, Zhang M-Z, Yin X-B, Peng R-W, Ming N-B (2003). Phys Rev E.

[32] Bruinsma O S L, van der Eerden J P (1995). Science and technology of crystal growth.

[33] Weng Y-Y, Si J-W, Gao W-T, Wu Z, Wang M, Peng R-W, Ming N-B (2006). Phys Rev E.

[34] Elechiguerra J L, Larios-Lopez L, Liu C, Garcia-Gutierrez D, Camacho-Bragado A, Yacaman M J (2005). Chem Mater.

[35] Galanakis I, Bihlmayer G, Bellini V, Papanikolaou N, Zeller R, Blügel S, Dederichs P H (2002). EPL.

[36] Marsili M (1992). J Phys A: Math Gen.

[37] Chazalviel J-N (1990). Phys Rev A.

